# Identification of pathways and genes associated with synovitis in osteoarthritis using bioinformatics analyses

**DOI:** 10.1038/s41598-018-28280-6

**Published:** 2018-07-03

**Authors:** Hui Huang, Jiaxuan Zheng, Ningjiang Shen, Guangji Wang, Gang Zhou, Yehan Fang, Jianping Lin, Jianning Zhao

**Affiliations:** 1 Department of Orthopaedic Surgery, Jinling Hospital(Nanjing General Hospital of Nanjing Military Region), The First School of Clinical Medicine, Southern Medical University(Guangzhou), 305 East Zhongshan Road, Nanjing, 210002 Jiangsu Province China; 2 Department of Orthopaedic Surgery, Hainan Provincial People’s Hospital, Haikou, 570311 Hainan Province China

## Abstract

Synovitis in osteoarthritis (OA) is a very common condition. However, its underlying mechanism is still not well understood. This study aimed to explore the molecular mechanisms of synovitis in OA. The gene expression profile GSE82107 (downloaded from the Gene Expression Omnibus database) included 10 synovial tissues of the OA patients and 7 synovial tissues of healthy people. Subsequently, differentially expressed gene (DEG) analysis, GO (gene ontology) enrichment analysis, pathway analysis, pathway network analysis, and gene signal network analysis were performed using Gene-Cloud of Biotechnology Information (GCBI). A total of 1,941 DEGs consisting of 1,471 upregulated genes and 470 downregulated genes were determined. Genes such as PSMG3, LRP12 MIA-RAB4B, ETHE1, SFXN1, DAZAP1, RABEP2, and C9orf16 were significantly regulated in synovitis of OA. In particular, the MAPK signalling pathway, apoptosis, and pathways in cancer played the most important roles in the pathway network. The relationships between these pathways were also analysed. Genes such as NRAS, SPHK2, FOS, CXCR4, PLD1, GNAI2, and PLA2G4F were strongly implicated in synovitis of OA. In summary, this study indicated that several molecular mechanisms were implicated in the development and progression of synovitis in OA, thus improving our understanding of OA and offering molecular targets for future therapeutic advances.

## Introduction

In terms of joint disease, osteoarthritis (OA) is the most common form of arthritis and is linked to a high rate of disability. Characterized by the degeneration and destruction of the arthrodial cartilage and hyperosteogeny, the predilection age group of OA is middle age^1^. It is well established that OA is not only a single cartilage lesion but also a lesion involving the entire articular cartilage, subchondral bone, and synovial membrane^2,3^.

For a long time, researchers who studied the pathogenesis mechanism of OA have focused on the pathological changes of articular cartilage and chondrocytes and have not paid much attention to the inflammatory changes of the synovium^4,5^. In particular, they even consider synovial inflammation a secondary change in the pathogenesis of osteoarthritis^6^. In addition, the intensity of research in recent years has revealed that the inflammatory mediators produced by synovial inflammation of joints can act not only on articular cartilage but also on some cytokines and proteases that regulate the metabolism of articular cartilage^7^. On the one hand, synovial inflammation leads to changes in articular cartilage structures and destroys and degrades the cartilage matrix. On the other hand, synovial inflammation exerts a physicochemical action on some cytokines and proteases, accelerating the process of joint degeneration^8^.

In OA, the synovium, which is the tissue that lines the joint capsule, frequently reveals inflammation signs, such as those of macrophages that infiltrate the synovium. Pro-inflammatory cytokines such as TNFα and IL1β are produced by these macrophages, activating fibroblast-like synoviocytes to release matrix-degrading enzymes and extra cytokines (IL8, IL6)^9^. These enzymes and cytokines impair the articular cartilage, leading to the discharge of damage-associated molecular patterns that also have pro-inflammatory functions, producing a perpetuating loop that causes chronic low-grade inflammation^10,11^. This inflammation is indicated to contribute to disease progression and disease phenotype^12^. Studying the fundamental pathological processes in the OA synovium would help in obtaining a treatment for this disease.

In the present study, we downloaded the microarray data of accession number GSE82107, which included 10 synovial tissues of osteoarthritis patients (OA) and 7 synovial tissues of healthy controls (HC)^13^. Then, we identified the differentially expressed genes (DEGs) between OA and HC samples to explore the molecular mechanisms of synovitis in osteoarthritis. In addition, we performed GO enrichment analysis, pathway analysis, pathway network analysis, and gene signal network analysis. This study findings may play significant roles in the genesis of OA and may serve as potential biomarkers in both the prognosis and diagnosis of OA.

## Results

### DEG identification

**Figure 1:**
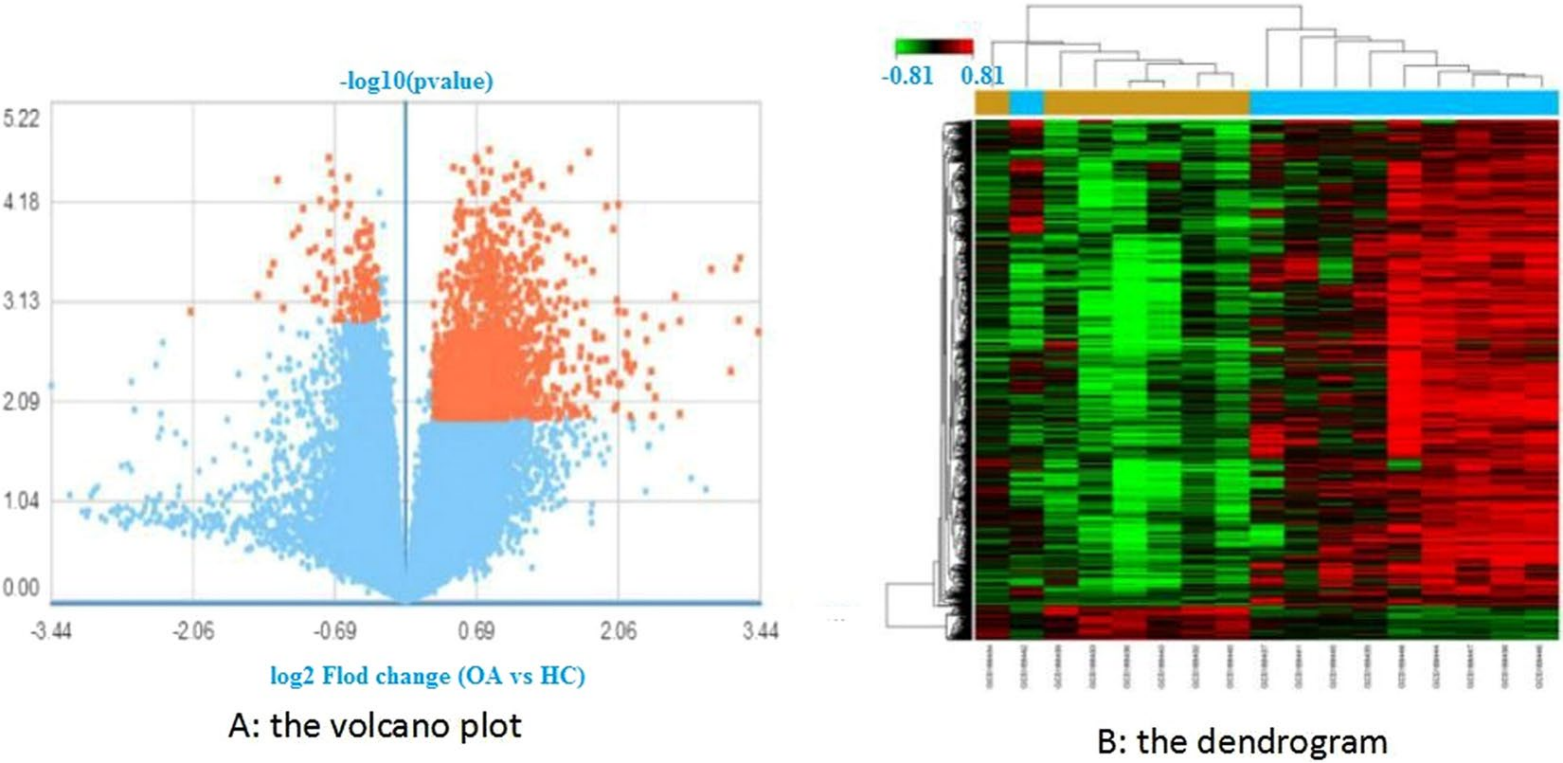
(**A**) The volcano plot. Orange represents DEGs; the downregulated DEGs are on the left side of the midline, and the upregulated DEGs are on the right. OA represents osteoarthritis patients; HC represents healthy controls. (**B**) The dendrogram. Profiles of DEGs in synovitis of OA. Data of mRNA were clustered using GCBI (P < 0.05). In total, 1941 DEGs were determined compared to the HC group (yellow). The OA group is blue, and the HC group is yellow.

The identification of DEGs is a statistical method of screening high-throughput genetic data and selecting genes with significant differences between samples^14^. The significance of DEG identification is Q < 0.05, P < 0.05 and a fold change >1.2. After data preprocessing, a total of 1,941 genes were determined to be differentially expressed in the OA samples compared to the HC samples. Among these DEGs, 470 genes were downregulated, and 1,471 were upregulated. The result is displayed in the volcano plot and the dendrogram (Fig. 1). The top 13 genes with the most significant expression were PSMG3, LRP12, unnamed, MIA-RAB4B, ETHE1, SFXN1, DAZAP1, RABEP2, C9orf16, HEMK1, SAC3D1, EIFIAD, and PSPC1 (Table 1).

**Table 1:** Top 13 genes with the most significant expression.

Gene symbol	Gene Description	P-value	Gene feature	Rank
PSMG3	Proteasome (prosome, macropain) assembly chaperone 3	1.9e-05	up	1
LRP12	Low density lipoprotein receptor-related protein 12	2e-05	up	2
—	unnamed	2.3e-05	down	3
MIA-RAB4B	MIA-RAB4B readthrough (NMD candidate)	2.3e-05	up	4
ETHE1	ethylmalonic encephalopathy 1	2.5e-05	up	5
SFXN1	sideroflexin 1	2.7e-05	up	6
DAZAP1	DAZ associated protein 1	2.9e-05	up	7
RABEP2	rabaptin, RAB GTPase binding effector protein 2	3e-05	up	8
C9orf16	chromosome 9 open reading frame 16	3.1e-05	up	9
HEMK1	HemK methyltransferase family member 1	3.1e-05	up	10
SAC3D1	SAC3 domain containing 1	3.2e-05	up	11
EIF1AD	eukaryotic translation initiation factor 1A domain containing	3.2e-05	up	12
PSPC1	paraspeckle component 1	3.3e-05	down	13

### Results of the GO enrichment analysis

In this paper, the GO enrichment analysis using the GCBI platform formed the basis for the obtained DEGs. By studying the enrichment degree of GO terms in the statistical analysis of DEGs, the biological processes that are most likely to be related to the DEGs were calculated, including the p-value and the FDR value of the GO term of the DEG. For the significance of the GO enrichment analysis, biological processes with P < 0.05 and FDR < 0.05 were selected as statistically significant biological processes. We developed enrichment analysis and significance analysis diagrams based on the GO enrichment analysis. After a comprehensive analysis, the top 10 biological processes were summarized, including: transcription DNA-dependent, protein transport, small molecule metabolic process, regulation of transcription, DNA-dependent, blood coagulation, cellular protein metabolic process, apoptotic process, negative regulation of transcription, DNA-dependent, gene expression, and small GTPase mediated signal transduction (Table 2).

**Table 2:** The top 10 biological processes of the gene ontology analysis. FDR, false discovery rate.

BP name	Enrichment score	P-value	FDR	Rank
transcription, DNA-dependent	2.67	5.23e-33	1.62e-29	1
protein transport	4.50	1.17e-23	1.82e-20	2
small molecule metabolic process	2.56	5.92e-22	6.12e-19	3
regulation of transcription, DNA-dependent	2.57	3.18e-21	2.47e-18	4
blood coagulation	3.52	6.8e-17	4.22e-14	5
cellular protein metabolic process	3.34	1.54e-16	7.95e-14	6
apoptotic process	2.88	1.07e-14	4.73e-12	7
negative regulation of transcription, DNA-dependent	3.24	1.42e-13	5.51e-11	8
gene expression	2.74	2.94e-13	1.01e-10	9
small GTPase mediated signal transduction	3.49	2.66e-12	8.24e-10	10

### Results of the pathway analysis and pathway network analysis

**Figure 2:**
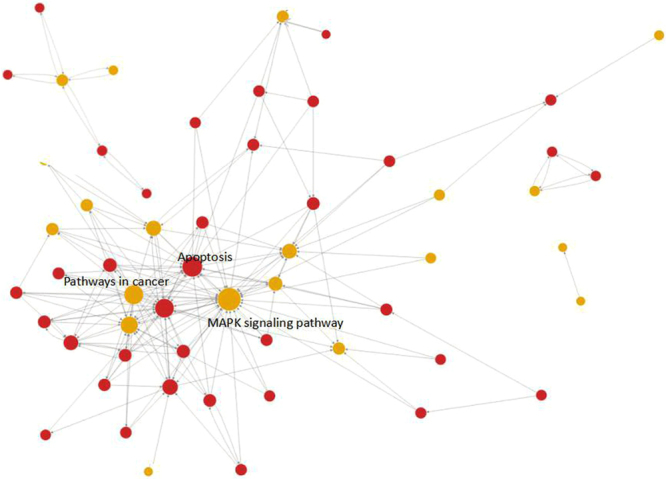
The constructed interaction network of pathways. The dots represent the pathway, and the size is represented by the degree value (the greater the value is, the more important the pathway is). Red represents upregulation, blue represents downregulation, and yellow represents both upregulation and downregulation. Arrows represent the relationship between upstream and downstream.

Through the pathway analysis of the DEGs, we can determine pathway items of the enriched DEGs and identify the cell pathway changes that may be related to the DEGs of different samples. Pathways with P < 0.05 were selected as statistically significant pathways. The pathway network analysis was used to analyse the signal transduction between significant pathways, which was found by the pathway analysis. In the network, the signalling pathway had more interactions, and therefore, it was more important. We identified a total of 117 pathways that were differentially regulated. These pathways included metabolic processes, lysosome, p53 signalling, viral carcinogenesis, Hepatitis B, HTLV-I infection, Vibrio cholerae infection, glycosylphosphatidylinositol (GPI)-anchor biosynthesis, protein processing in endoplasmic reticulum, and N-Glycan biosynthesis. In the pathway network analysis, a total of 56 pathways, including 37 upregulated pathways and 19 up/downregulated pathways, were identified. We identified 156 relationships between all determined pathways (Fig. 2). In the pathway relation network analysis (Table 3), there were 10 pathways with a degree score ≥10, including the MAPK signalling pathway (degree score = 33, pathway feature: up and down), apoptosis (degree score = 25, pathway feature: only up) and cancer pathways (degree score = 23, pathway feature: up and down).

**Table 3:** The top 10 degree scores of the analysis methodology of the pathway relation network. Greater degree values indicate pathways with greater importance.

Pathway name	Outdegree	Indegree	Degree	Pathway feature
MAPK signalling pathway	5	28	33	up|down
Apoptosis	3	22	25	up
Pathways in cancer	23	0	23	up|down
Cell cycle	3	19	22	up
p53 signalling pathway	2	16	18	up|down
Wnt signalling pathway	6	8	14	up
Focal adhesion	7	6	13	up|down
ErbB signalling pathway	6	6	12	up
Cytokine-cytokine receptor interaction	0	12	12	up|down
Jak-STAT signalling pathway	5	5	10	up|down

These results indicated that the degree values of the MAPK signalling pathway, apoptosis, and cancer pathways were the highest. The nature of their relationships remains unknown. Based on the information listed in the GCBI, the cancer pathways were upstream of the MAPK signalling pathway, which was in turn upstream of the apoptosis pathway. In other words, the pathways downstream of the cancer signalling pathways were the apoptosis and MAPK signalling pathways, while the pathways upstream of the apoptosis signalling pathway were the cancer and MAPK signalling pathways.

### Results of gene signal network analysis

**Figure 3:**
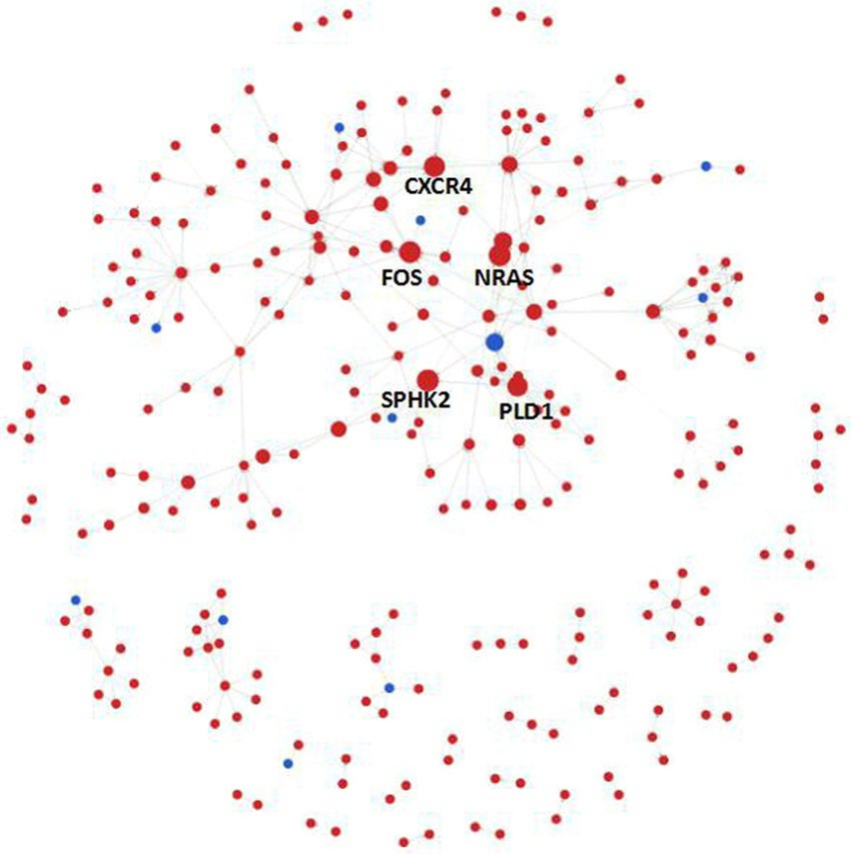
The constructed interaction network of genes. The dots represent hub genes, and the size represents the betweenness centrality value (the greater the value is, the more important the gene is). Red represents upregulation and blue represents downregulation. Arrows represent the relationship between upstream and downstream.

**Figure 4:**
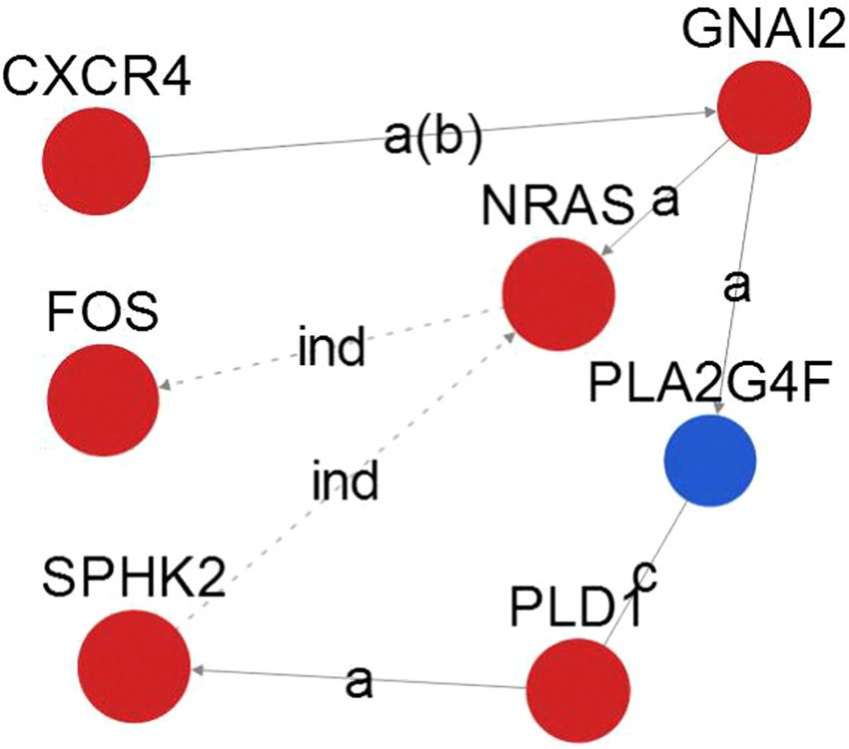
The dots represent genes, and the size represents the degree value (the greater the value is, the more important the pathway is). Red represents upregulation and blue represents downregulation. Arrows represent the relationship between upstream and downstream. The dotted line indicates the indirect action.

The gene signal network analysis constructs the network of gene interactions, based on the interaction between the proteins and the proteins in KEGG database. In the gene signal network analysis, a total of 257 hub nodes (hub genes), including 246 upregulated genes and 11 downregulated genes, were identified. In addition, there were 337 relationships between all identified genes (Fig. 3). The top 15 hub nodes with a higher betweenness centrality value were screened. These hub genes included NRAS, SPHK2, FOS, CXCR4, PLD1, GNAI2, PLA2G4F, PIK3R2, PRKCA, ASAH1, CCL5, CCL3, UGCG, RELA, and ITGB5 (Table 4). We subsequently selected the top seven hub genes and analysed the upstream and downstream relationships between them (Fig. 4).

**Table 4:** The top 15 betweenness centrality values of the gene signal network analysis. Greater betweenness values indicate genes with greater importance.

Gene symbol	Gene feature	Gene Description	Betweenness
NRAS	up	neuroblastoma RAS viral (v-ras) oncogene homolog	2509.5
SPHK2	up	sphingosine kinase 2	2509
FOS	up	FBJ murine osteosarcoma viral oncogene homolog	2478.83
CXCR4	up	chemokine (C-X-C motif) receptor 4	2313.33
PLD1	up	phospholipase D1, phosphatidylcholine-specific	2168.5
GNAI2	up	guanine nucleotide binding protein (G protein), alpha inhibiting activity polypeptide 2	1767.17
PLA2G4F	down	phospholipase A2, group IVF	1693
PIK3R2	up	phosphoinositide-3-kinase, regulatory subunit 2 (beta)	1375
PRKCA	up	protein kinase C, alpha	1328
ASAH1	up	N-acylsphingosine amidohydrolase (acid ceramidase) 1	1260
CCL5	up	chemokine (C-C motif) ligand 5	1116.67
CCL3	up	chemokine (C-C motif) ligand 3	1057.67
UGCG	up	UDP-glucose ceramide glucosyltransferase	1008
RELA	up	v-rel avian reticuloendotheliosis viral oncogene homologue A	999
ITGB5	up	integrin, beta 5	969

## Discussion

OA is a chronic joint disease characterized by degeneration of articular cartilage and hyperosteogeny. OA inflammatory synovitis of the knee has been considered a secondary reaction to the stimulation of articular cartilage remnants and debris and is the main cause of joint swelling and joint pain^15^. However, recent studies have shown that a separate relationship exists between the degree of OA synovitis and the stimulation of articular cartilage remnants and debris, for which the cause is unknown. The results of OA arthroscopic synovial tissue examination of moderate-to-severe knee joint OA have also shown that there are pathological changes in synovium, which are not simply secondary reactions. OA synovitis may be caused by many unexplained factors, and the stimulation of articular cartilage remnants and debris is only one of the mechanisms that cause knee OA clinical symptoms. In the late stage of OA, the synovium in the articular cavity has been stimulated for a long time, showing villous hyperplasia and an erosion of the articular cartilage surface, destroying cartilage and bone, which accelerates the progression of the disease and worsens the symptoms^16^. Therefore, the clarification of signalling pathways and signal molecules that abnormally change in OA synovial cells is of great theoretical significance and informs clinical guidance for an in-depth understanding of the occurrence and development of OA and the role of synovial cells in this process.

We screened the DEGs implicated in the development of synovitis in OA using bioinformatics analysis, in an attempt to reveal the pathogenesis of synovitis. The results indicated that thousands of genes in OA synovial tissues have undergone molecular biological changes, with a total of 1,941 DEGs determined, including 1,471 upregulated and 470 downregulated genes, compared with normal synovial tissues. The results also indicated that the number of the upregulated genes was significantly higher than the downregulated genes, indicating that the upregulation of OA synovial lesions was dominant, accounting for 75.79%. We further screened 13 genes with the most significant expression changes, namely, PSMG3, LRP12, unnamed, MIA-RAB4B, ETHE1, SFXN1, DAZAP1, RABEP2, C9orf16, HEMK1, SAC3D1, EIFIAD, and PSPC1. Among these, PSMG3 exhibited the highest expression (gene ID: 84262; updated on July 15, 2015; gene type: protein-coding; species: Homo sapiens; alias: C7orf48 or PAC3; other sources: Ensemble: ENSG 00000157778, HPRD: 14404, and Vega: OTTHUMG00000119043; related diseases: neoplasms, autistic disorder, fibrosis, growth disorders, liver diseases, shock; related IncRNA: LINC01561 and ZRANB2-AS2; targeted miRNA: hsa-miR-92a-3p). The expression of PSMG3 has been reported to be upregulated in a variety of cancer tissues^17–20^. Our results indicated that the expression of PSMG3 was also upregulated in the OA synovial tissues, highlighting the tumour-like properties of the OA synovial tissue.

The GO enrichment analysis revealed that the DEGs were mainly involved in pathways related to transcription DNA-dependent, protein transport, small molecule metabolic process, regulation of DNA-dependent transcription, blood coagulation, cellular protein metabolic process, apoptotic process, negative regulation of DNA-dependent transcription, gene expression, and small GTPase mediated signal transduction. These results confirmed that the pathogenesis of synovitis in osteoarthritis is the result of multiple factors and genes that, directly or indirectly, lead to the occurrence and development of synovitis in osteoarthritis through mutual gene and network regulation. In the present study, we were particularly interested in the apoptotic process (Accession GO:0006915). Apoptosis is a common physiological and pathological phenomenon in the process of biological growth and development. It is the process of self-destruction of living tissue cells that is regulated by genes or other factors. Apoptosis is implicated in various pathological functions, including autoimmune diseases, viral infections, occurrence and regression of malignant tumours, and cardiovascular and cerebrovascular diseases. Our present results indicated a specifically high regulation of the apoptotic process in the OA synovial tissue. Based on this result, we suggest that the development of synovitis of OA is related to autoimmunity and tumour-like lesions^21^. Further research is still needed to validate our findings.

In the pathway network analysis, the MAPK, apoptosis, and cancer signalling pathways are the three pathways with the highest degree value, showing that these signalling pathways may play the most important role in osteoarthritis synovitis. Among them, the MAPK pathway is the most important signal transduction system that mediates osteoarthritis cartilage injury, which binds to receptors on the cell membrane by mainly utilizing the specificity of affected joints, inflammatory factors (such as IL-1 and TNF-α), growth factors, and activating intracellular MAPKs signal transduction pathways, causing a series of reactions, such as the increased expression of matrix MMPs (MMP-1, MMP-3, MMP-13), chondrocyte apoptosis, cartilage destruction and so on^22^. Our study also suggests that the MAPK pathway also plays an important role in osteoarthritis synovitis. We hypothesized that MAPK-related protein kinase inhibitors not only protect the OA cartilage but also play an important role in the control of synovitis. The procedure of apoptosis is extremely complicated; it is a significant mechanism that regulates the normal development of the body and is involved in a series of regulatory factors that play an important role in maintaining cell homeostasis. The occurrence, development, and treatment of tumours are also regulated by many apoptosis regulating proteins^23,24^. Our results indicated that the apoptosis signalling pathway was upregulated in the OA synovial tissue, suggesting that apoptosis is implicated in OA synovial lesions and may also promote cartilage cells apoptosis. In addition, we also found a high regulation of cancer signalling pathways in the synovial tissues, which corroborates our notion that OA synovial tissue lesions have tumour-like properties.

In the gene signal network analysis, we selected the 15 most important genes, which played a key role in OA synovial lesions, for further analysis. It was essential to reveal the molecular biological mechanism in synovial lesions of OA and even in OA alone. These genes were implicated in the MAPK signalling pathway, cancer signalling pathways, Wnt signalling pathway, and cytokine-cytokine receptor interaction, among others. The results were basically the same as those from the pathway network analysis. The biological processes involved were protein transport, small molecule metabolic process, blood coagulation, cellular protein metabolic process, and apoptotic process, among others and were consistent with the GO functional analysis. The gene NRAS is an N-ras oncogene encoding a membrane protein that shuttles between the Golgi apparatus and the plasma membrane. This shuttling is regulated through palmitoylation and depalmitoylation by the ZDHHC9-GOLGA7 complex. The encoded protein, which has an intrinsic GTPase activity, is activated by a guanine nucleotide-exchange factor and inactivated by a GTPase activating protein. Mutations in this gene have been associated with somatic rectal cancer, follicular thyroid cancer, autoimmune lymphoproliferative syndrome, Noonan syndrome, and juvenile myelomonocytic leukaemia. In the present study, we found that NRAS played an important role in synovitis in OA. Thus, further research is warranted to elucidate the exact effect of NRAS in the OA synovium. The FOS gene is an immediate early gene. When it is stimulated, the FOS gene in the nucleus is activated and FOS protein is synthesized. After phosphorylation, FOS protein returns to the nucleus and forms a complex transcription activator protein 1 with the jun protein, encoded by another family of proto-oncogene c-jun, which binds to the target gene TPA response element to activate transcription. Due to its target gene diversity, transcription activator protein 1 is closely related to the important physiological processes of cells, inflammation and other diseases. FOS not only has an important influence on the proliferation and differentiation of chondrocytes but is also strongly associated with cartilage inflammation. FOS acts as an important messenger in the process of cartilage inflammation, which mediates the inflammatory response of the interleukin family (such as interleukin 17 and interleukin 1β), calcium-containing crystals, and mechanical stimulation^25^. Our research suggests that the FOS gene plays a key role in the synovitis of osteoarthritis, and the NRAS gene indirectly acts on FOS, which provides the theoretical basis and ideas for further experimental study.

The high-throughput and high-sensitivity detection of the gene chip is a double-edged sword. In practice, the gene expression profiles of animal tissues and cells are not homogenous. There are certain variations among different individuals or cell lines, and the expression profile is also highly susceptible to changes in experimental conditions. Small differences in RNA extraction and cDNA reverse transcription and other steps often cause changes in the expression of different genes, which may make it difficult to determine the biological significance of a large number of genes with positive changes in the microarray assay. On the other hand, actual chip probe hybridization often produces a large number of false positives and false negatives. For the accuracy of single gene determination, the high-throughput microarray is not as accurate as a low-throughput Northern blot, real-time PCR and other technologies. It should also be noted that the other limitation of the application of microarrays is that the changes in determined mRNA levels are only intermediate products of gene expression and are not functional proteins, which are far from directly explaining the mechanisms that functional proteins are mainly involved with in a variety of physiological and pathological changes at the cell and tissue level. Moreover, even if the microarray profile shows different genes with significant changes and the causal relationship among them, the exact judgement cannot rely solely on the microarray technology, and it needs further study using corresponding experimental techniques^26^. Therefore, DNA microarrays should be mainly positioned to use the high-throughput approach to observe the overall genome changes under specific experimental conditions to determine effective clues from the complex expression profile to develop the qualitative experiments for further study. It should also be noted that compared with single or few gene expression detected by traditional molecular biology techniques, the specific mRNA expression patterns of expression profiles observed in microarrays can reflect and predict the corresponding biological mechanisms more comprehensively. Before the popularization of proteome technology, DNA microarray technology was the most effective method for studying gene expression at the genome level^27^.

In summary, our present study provided a comprehensive bioinformatics analysis of DEGs, biological processes terms, hub genes, and pathways, which might be associated with OA synovial inflammation. The present results could facilitate improving our comprehension of the underlying molecular mechanisms of OA synovial inflammation. Genes such as NRAS, SPHK2, FOS, CXCR4, PLD1, GNAI2, and PLA2G4F, and their related biological process terms and pathways, such as apoptosis, MAPK signalling pathway, and cancer signalling pathways, may represent potential targets for OA treatment and diagnosis. In the present data analysis, the sample size was small, and the samples were selected from one platform. This may result in a high rate of false positive results. Additional experimental and genetic studies with a larger sample size are still guaranteed to confirm our present findings.

## Methods

### Gene-Cloud of Biotechnology Information (GCBI)

GCBI (Shanghai, China, https://www.gcbi.com.cn) is a platform combining various kinds of sample information, genetic information, research findings, data algorithms and bioinformatics to generate a “gene knowleDEG base”, which encompasses biology, mathematics, informatics, medicine, computer science, graphics and other disciplines. Over 120 million copies of genomic samples, approximately 90,000 copies of tumour samples and over 17 million copies of genetic information are included in the GCBI platform^28–32^. In this study, we used GCBI to identify DEGs between OA and HC samples and perform GO enrichment analysis, pathway analysis, pathway network analysis, and gene signal network analysis.

### Affymetrix microarray data

In the National Centre of Biotechnology Information (NCBI) Gene Expression Omnibus database (GEO, http://www.ncbi.nlm.nih.gov/geo/), the key words of “Synovial biopsies of osteoarthritis patients” were used, and the gene chip data sets GSE82107 (https://www.ncbi.nlm.nih.gov/geo/query/acc.cgi?acc = GSE82107), which were submitted by Broeren, *et al*. on June 1, 2016, were obtained. GSE82107, which contained a total of 17 samples, including 10 synovial tissues of OA and 7 synovial tissues of HC, was based on the platform of GPL570 [HG-U133_Plus_2] Affymetrix Human Genome U133 Plus 2.0 Array^13^. For microarray analysis, synovial samples were obtained by surgery or via fine-needle arthroscopy from 10 OA patients and 7 controls.

### Design of the analysis process

**Figure 5:**
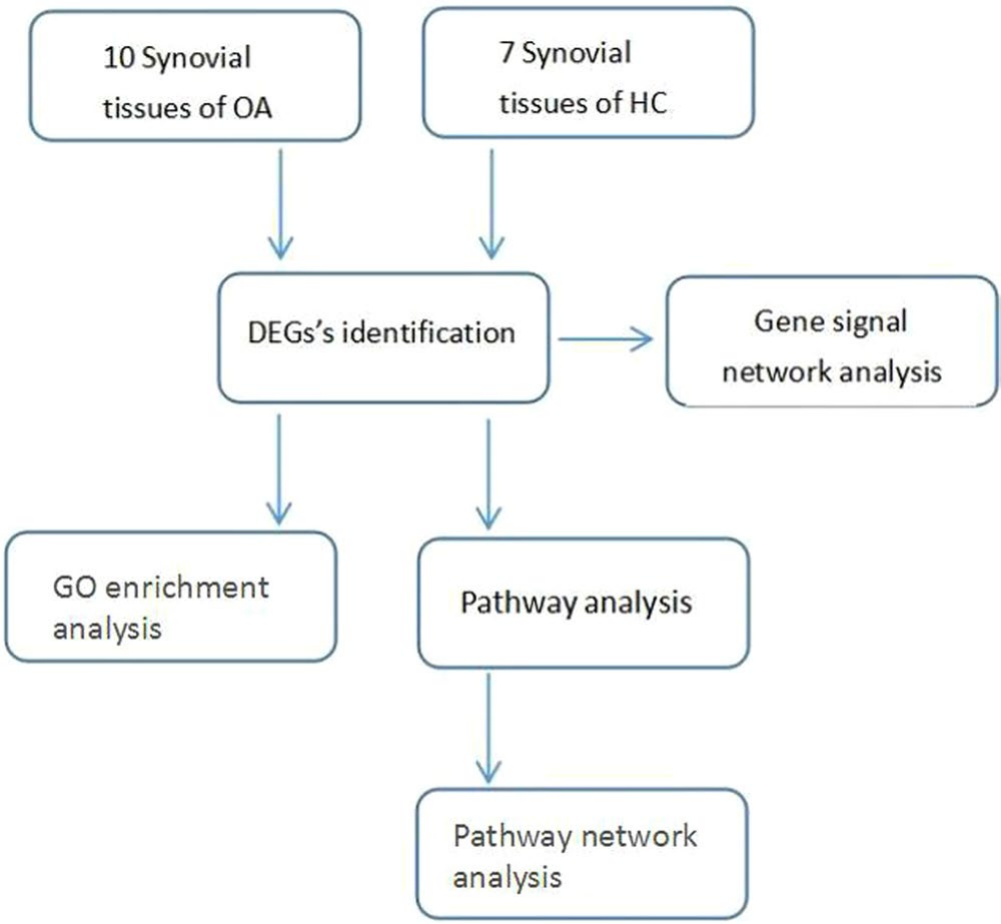
Flow diagram of the study design.

Sample gene chip information was input to the Gene-Cloud of Biotechnology Information (GCBI) analysis platform (https://www.gcbi.com.cn/gclib/html/index) for data analysis. First, the 17 samples were divided into 10 OA cases (OA group) and seven control cases (HC group). Second, the differentially expressed genes (DEGs) were identified. Subsequently, we performed GO enrichment analysis, pathway analysis, and gene signal network analysis. Finally, pathway network analysis was performed based on the results of the pathway analysis. The flow diagram of our study design is shown in Fig. 5.

### Identification of DEGs

A data stability test was performed on the microarray data of GSE82107 using the GCBI platform, and the microarray data of GSE82107 was filtered by two independent samples (Q < 0.05, P < 0.05, fold change >1.2) to screen out the difference between OA samples and HC samples’ expression of genes.

### GO enrichment analysis and pathway analysis

GO, the abbreviation of gene ontology, is a tool that unifies biology by collecting structured, defined and controlled vocabulary for a large scale of genes annotation. Through GO enrichment analysis, it is possible to understand the biological functions of differential gene enrichments^33^. Pathway refers to metabolic pathways. Pathway analysis of DEGs can be used to understand the significantly altered metabolic pathways under experimental conditions and is particularly important in mechanistic studies^34^. We performed GO enrichment analysis (P < 0.05, FDR < 0.05) and pathway analyses (P < 0.05) of the DEGs using the GCBI platform.

### Pathway network analysis and gene signal network analysis

Pathway network analysis is based on the upstream and downstream relationship of signal pathways in the KEGG database, and the interaction network diagram of pathway research is constructed. The pathway network analysis can help to determine the pathway that has a regulating effect on the top stream and on the lowest stream at the same time. By comprehending the relationship between pathways, a deeper understanding of signal pathways can be ascertained^35^. A large number of studies have shown that the expression of genes is affected by each other. This interactive and mutually restrictive relationship constitutes a complex network of gene expression and regulation. The gene signal network deconstructs the KEGG database, which breaks through the limit of acquiring the interactions of between genes in a single pathway. Therefore, a gene signal network can obtain a protein’s upstream or downstream proteins through the entire KEGG pathway database^36^. We performed pathway network analysis and gene signal network analysis to determine hub pathways and genes using the GCBI platform.
